# Amoxicillin Haptenation of *α*-Enolase is Modulated by Active Site Occupancy and Acetylation

**DOI:** 10.3389/fphar.2021.807742

**Published:** 2022-01-13

**Authors:** Juan M. González-Morena, Francisco J. Sánchez-Gómez, Yolanda Vida, Ezequiel Pérez-Inestrosa, María Salas, María I. Montañez, Alessandra Altomare, Giancarlo Aldini, María A. Pajares, Dolores Pérez-Sala

**Affiliations:** ^1^ Department of Structural and Chemical Biology, Centro de Investigaciones Biológicas Margarita Salas (CSIC), Madrid, Spain; ^2^ Dpto. Química Orgánica, Universidad de Málaga-IBIMA, Málaga, Spain; ^3^ Centro Andaluz de Nanomedicina y Biotecnología-BIONAND, Parque Tecnológico de Andalucía, Málaga, Spain; ^4^ Allergy Research Group, Instituto de Investigación Biomédica de Málaga-IBIMA, Allergy Unit, Hospital Regional Universitario de Málaga, Málaga, Spain; ^5^ Allergy Research Group, Instituto de Investigación Biomédica de Málaga-IBIMA, Andalusian Centre for Nanomedicine Biotechnology-BIONAND, Parque Tecnológico de Andalucía, Málaga, Spain; ^6^ Department of Scienze Farmaceutiche, Universita degli Studi di Milano, Milan, Italy

**Keywords:** beta-lactam antibiotics, protein modification by drugs, mass spectrometry, posttranslational modification, acetylation, allergic responses to drugs

## Abstract

Allergic reactions to antibiotics are a major concern in the clinic. *ß*-lactam antibiotics are the class most frequently reported to cause hypersensitivity reactions. One of the mechanisms involved in this outcome is the modification of proteins by covalent binding of the drug (haptenation). Hence, interest in identifying the corresponding serum and cellular protein targets arises. Importantly, haptenation susceptibility and extent can be modulated by the context, including factors affecting protein conformation or the occurrence of other posttranslational modifications. We previously identified the glycolytic enzyme *α*-enolase as a target for haptenation by amoxicillin, both in cells and in the extracellular milieu. Here, we performed an *in vitro* study to analyze amoxicillin haptenation of *α*-enolase using gel-based and activity assays. Moreover, the possible interplay or interference between amoxicillin haptenation and acetylation of *α*-enolase was studied in 1D- and 2D-gels that showed decreased haptenation and displacement of the haptenation signal to lower pI spots after chemical acetylation of the protein, respectively. In addition, the peptide containing lysine 239 was identified by mass spectrometry as the amoxicillin target sequence on *α*-enolase, thus suggesting a selective haptenation under our conditions. The putative amoxicillin binding site and the surrounding interactions were investigated using the *α*-enolase crystal structure and molecular docking. Altogether, the results obtained provide the basis for the design of novel diagnostic tools or approaches in the study of amoxicillin-induced allergic reactions.

## 1 Introduction

Adverse and hypersensitivity reactions to drugs used in clinical treatments constitute a severe clinical problem and may result in patient’s death. The most common among them are due to the administration of antibiotics, and were firstly observed soon after the start of penicillin use around 1940 (Blumenthal et al., 2019). According to a recent review, from 5 to 15% of clinical records on drug allergy report allergic reactions to penicillin or other *ß*-lactam antibiotics (Blumenthal et al., 2019), therefore representing a major subject of concern. The frequent use of amoxicillin (AX), whether administered alone or in combination with clavulanic acid, has increased the IgE-mediated allergic responses to this antibiotic and, thus, an interest in the proteins and epitopes triggering these effects has emerged. One of the mechanisms by which these epitopes are formed involves protein covalent modification by the antibiotics, a process known as haptenation. Mass spectrometry studies have been carried out to uncover AX-haptenated proteins in serum (Ariza et al., 2012), in cells (Ariza et al., 2014; Sánchez-Gómez et al., 2017) and in extracellular fractions (Sánchez-Gómez et al., 2017). Interestingly, modification of the glycolytic enzyme *a*-enolase by AX or its biotinylated form (AX-B) has been found both in lysates and the conditioned medium of cells treated with either form of the antibiotic (Sánchez-Gómez et al., 2017). Moreover, extracellular vesicles and the extracellular soluble protein fraction of these cultures contain AX-haptenated *a*-enolase (Sánchez-Gómez et al., 2017).

Human *α*-enolase is a 47 kDa protein encoded by the *ENO1* gene located in chromosome 1, which also contains an internal translation initiation site that leads to a 95% identical splicing form of 37 kDa named MBP-1 (Subramanian and Miller 2000). Two additional enolase proteins, *ß* and *γ,* 83% homologous to *α*-enolase are encoded by *ENO3* and *ENO2* genes, respectively (reviewed in (Pancholi 2001; Terrier et al., 2007). The three enolase isoenzymes present developmental and tissue dependent expression patterns. Therefore, although *ENO1* is highly conserved from microorganisms to mammals (Pancholi 2001), the gene is not considered among housekeeping genes. *α*-Enolase oligomerizes into homo- or hetero-dimers, the latter by association with *ß*- and *γ*-enolases (reviewed in (Pancholi 2001; Terrier et al., 2007). Dimerization, as well as binding of two Mg^2+^ ions per subunit, is required for *α*-enolase glycolytic activity (reviewed in (Pancholi 2001). Nevertheless, the enzyme can be activated by other cations, including Zn^2+^, Mn^2+^, Fe(II)^2+^, Cd^2+^, Co^2+^, Ni^2+^, Sm^3+^ or Tb^3+^. Among them, Zn^2+^ binding is much stronger than Mg^2^ binding (Brewer and Ellis 1983). The main subcellular location of *α*-enolase is the cytoplasm where homo-dimers have been detected. However, the enzyme has been also found in the nucleus, extracellular vesicles and as homo- and hetero-oligomers (*αγ*) at the cell membrane.

Cytoplasmic *α*-enolase participates in glycolysis and gluconeogenesis catalyzing 2-phopho-D-glycerate (2-PG) dehydration into phosphoenolpyruvate and the reverse reaction, respectively (Ji et al., 2016). However, the diverse distribution pattern of *α*-enolase correlates with moonlighting activities that require translocation to different cellular compartments or its secretion to the extracellular millieu. These additional *α*-enolase functions include: 1) growth control; 2) hypoxia tolerance; 3) activation of the fibrinolytic system, acting as a plasminogen receptor and activator when present at the surface of cells or bacteria (Miles et al., 1991; Serek et al., 2021); 4) stimulation of immunoglobulin production (Sugahara et al., 1992); 5) acting as autoantigen in autoimmune events (e.g., rheumatoid arthritis or Hashimoto encephalopathy); 6) transcriptional regulation (in the case of the nuclear MBP-1 form) (Feo et al., 2000; Maranto et al., 2015); 7) involvement in parasite proliferation (e.g. *Toxoplasma gondii* or *Plasmodium genus*) (Ferguson et al., 2002; Foth et al., 2008); 8) binding to tRNA in yeast (Shchepachev et al., 2019); 9) functioning as chaperone, the sequence of HSP48 corresponds to that of *α*-enolase (Iida and Yahara 1985); 10) exerting a structural function in the lens as a monomeric *τ*-crystallin (Wistow and Piatigorsky 1987; Wistow et al., 1988); and 11) contributing to allergic responses (e.g., food allergies (Apostolovic et al., 2014)). Several reports have identified the existence of natural variants such as N^177^K (dbSNP: rs11544513) and P^325^Q (dbSNP: rs11544514), as well as mutations linked to functional impairment. Among the latter, association of two methionine to isoleucine mutations (M^94^I and M^97^I) impairs MBP-1 production (Subramanian and Miller 2000), whereas association of two leucine to alanine mutations (L^388^A and L^384^A) lead to decreased transcriptional repression activity (Ghosh et al., 1999).

Two X-ray crystal structures of the whole human *α*-enolase are available in the PDB database (2PSN and 3B97), which lack the initial methionine residue. These structures show the different topologies of N-terminal (1–138) and C-terminal domains (139–432), the former having *β*_3_ *α*_4_ structure while the latter forms a (*αβ*)_8_ barrel (Kang et al., 2008). The active site locates at the C-terminal domain with catalytic residues placed at both opposite sides of a cavity that also involves the C-terminal ends of the *ß*-strands of the barrel. However, the N-terminal region is also important for catalysis as demonstrated by absence of activity of the MBP-1 form that lacks the initial 96 residues of *α*-enolase. Other regions of the protein are required for substrate (S^370^-S^373^), DNA (E^96^-T^236^), plasminogen binding (F^250^-Y^256^, A^405^-K^434^) or for Myc repression (M^97^-T^237^). Monomers associate in antiparallel fashion to form the dimer, so that the N-terminal of subunit A locates in front of the C-terminal of subunit B (Kang et al., 2008). According to structural data, binding of Mg^2+^ to one site induces a conformational change that allows substrate binding to the active site, whereas binding to the second site is required for catalysis (Faller et al., 1977).

Several proteoforms of *α*-enolase can be detected by 2D-electrophoresis as distinct spots (Lee et al., 2003). These proteoforms result from a variety of posttranslational modifications (PTMs) (e.g., removal of the first methionine) that in several cases have been linked to the role of *α*-enolase in pathology. In fact, analysis of data from 2D-based proteomic studies of human, mouse and rat tissues under a variety of conditions show *α*-enolase among the most differentially expressed proteins (Petrak et al., 2008). Additionally, *α*-enolase has been involved in several pathophysiological processes and anti-enolase antibodies found in several malignancies (Tomaino et al., 2011), microorganism infections, early stages of liver fibrosis (Peng et al., 2013), autoimmune diseases (reviewed in (Pancholi 2001)) and in allergic responses (Simon-Nobbe et al., 2000). In some cases, the autoantibodies recognized specific proteoforms that are phosphorylated (e.g., phospho-S^419^ in pancreatic cancer (Tomaino et al., 2011)) or contain acetylation sites (e.g., the peptide D^98^-M^165^ identified in autoimmune lupus nephritis (Bruschi et al., 2014)). *α*-Enolase from diverse species is also an important allergen and PTMs are also recognized in food allergies such as red meat allergy, in which the carbohydrate galactose-*α*-1,3-galactose (*α*-Gal) epitope is recognized in *α*- and *ß*-enolases that, due to their high thermal stability, preserve the structural features of their natural epitopes even after cocking (Apostolovic et al., 2014). Thermal stability is also observed in enolases of other origins, although it does not seem to be a general property of these enzymes (Kustrzeba-Wójcicka and Golczak 2000; Ji et al., 2016). Anti-citrullinated and anti-carbamylated antibodies found in rheumatoid arthritis recognize *α*-enolase ((Reed et al., 2016; Li et al., 2017) and reviewed in (Klareskog et al., 2008; Wegner et al., 2010)), further confirming the role of PTMs in the generation of epitopes on the protein structure. Oxidized forms of *α*-enolase have been also found as targets of autoantibodies in Hashimoto encephalopathy (Ochi et al., 2002) and proposed to be the epitopes recognized in liver fibrosis (Peng et al., 2013). Phosphorylation and citrullination of *α*-enolase increase the affinity for the Major Histocompatibility Complex, hence favoring the formation of the corresponding complex and its presentation to T-cells (Mohammed et al., 2008).

Mapping of anti-enolase IgE epitopes generated during allergic responses against *Cladosporium herbarum* and *Alternaria alternate* identify the C^120^-V^189^ peptide (Simon-Nobbe et al., 2000), which spans the whole diameter of the protein structure (from one surface to the opposite), exposing amino acid lateral chains at several points. According to molecular modeling, IgE binding most probably involves A^124^-L^130^, D^136^-P^143^, and R^163^, although mutagenesis studies later discarded K^141^ as the main epitope (Simon-Nobbe et al., 2000). The presence of autoantibodies is also used to distinguish mild from severe cases of asthma (Nahm et al., 2006); interestingly, severe asthma is often associated with hypersensitivity to aspirin (Lassalle et al., 1993). Several epitopes on *α*-enolase are also recognized by antibodies found in cancer associated retinopathy (Adamus et al., 1996; Adamus et al., 1998), the specific epitope recognized by patient’s sera encompassing residues R^56^-S^63^. Another important epitope is located on peptide D^53^-Q^87^ and recognized by both autoantibodies of cancer associated retinopathy and endometriosis patients (Walter et al., 1995). Therefore, allergen databases such as the AllFam database, created to identify common structural features of allergens (Radauer et al., 2008), include enolases of different species. In fact, the allergen families were shown to comprise 184 Pfam structural domains, a result that restricts the apparent structural variety of epitopes to about 5% of structural protein families. Moreover, this analysis identified TIM barrels (*αβ*)_8_ as the most frequent domain type (Radauer et al., 2008), which is the topology found in the *α*-enolase C-terminal domain (PF00113) (Kang et al., 2008).

Altogether this information on the role of *α*-enolase as an allergen and the epitopes related to allergic reactions highlight the importance of PTMs in its antigenicity. However, few data regarding *α*-enolase modification by drugs and the implication of haptenation in allergy are available, and, hence, studies focused on its understanding are needed. Here, we have carried out an in depth *in vitro* characterization of *α*-enolase haptenation and identification of the AX-modified residues, as well as an analysis of its putative interplay or interference with additional PTMs targeting similar residues.

## 2 Materials and Methods

### 2.1 Materials

Amoxicillin (Clamoxyl®) was from GlaxoSmithKline and biotinylated amoxicillin (AX-B) was synthesized as previously described (Ariza et al., 2014). Electrophoresis products were from BioRad. Sulfo-NHS acetate (SNA) was obtained from ThermoFisher Scientific. Acetylsalicylic acid, penicillin G, trypsin inhibitor, NaF, and sodium orthovanadate were from Sigma. Pefablock, aprotinin, leupeptin and pepstatin A protease inhibitors were products of Roche. Biotinylated bovine serum albumin and BCA for protein quantification were obtained from Pierce.

### 2.2 Haptenation of *α*-Enolase by *ß*-Lactam Antibiotics in Solution

Haptenation reactions (10 μl) usually contained 2.12 μM recombinant *α*-enolase (1 μg; P06733, Acris AR09287PU-N) and a 10-fold molar excess of AX or AX-B (21.2 µM) in PBS and were carried out for 1 h at 37°C, unless otherwise indicated. Exceptions to this rule were as follows: 1) incubations with 2.12–21.2 μM AX-B lasted for 2 h; 2) for 2D-electrophoresis 4 µg of *α*-enolase were used; 3) preincubations with AX lasted for 2 h, before AX-B addition (2.12 μM) for 2 h more; 4) preincubations with 2.12 mM 2-PG, 0–20 mM MgCl_2_, or the combination of 2.12 mM 2-PG plus 10 mM MgCl_2_ for 1 h at 37°C preceded AX-B addition; 5) protein denaturation was carried out with 7 M urea or at 95°C for 10 min before AX-B addition; 6) for MALDI-TOF analysis 1-fold (2.12 μM) or 10-fold (21.2 μM) molar excess AX was used; 7) for ESI-MS, incubations with AX lasted 3 h; and 8) for identification of the haptenated residues by LC-ESI-MS/MS incubations included 2.12 mM AX and lasted 16 h. Penicillin G haptenation was carried out with 1 μg *α*-enolase and different antibiotic concentrations (20 μM-2 mM) for 1 h at 37°C.

### 2.3 *In vitro α*-Enolase Acetylation

Recombinant *α*-enolase (1 μg; 2.12 μM final concentration) was incubated with 18-, 36-, 90-, and 180-fold molar excess sulfo-NHS-acetate (SNA) or with 0.1–20 mM acetylsalicylic acid for 1 h at room temperature. Reactions were stopped by addition of equivalent amounts of Tris, which provides amino groups to compete with those of the protein as acetylation targets. The putative impact of acetylation on AX-B modification was analyzed by incubation of SNA acetylated *α*-enolase proteoforms with 21.2 μM AX-B for an additional hour at 37°C. Samples of the reactions were processed by SDS-PAGE and electrotransference. For 2D-electrophoresis, acetylation reactions contained *α*-enolase (4 μg) and a 180-fold molar excess of SNA (0.4 μg) or 4 μg each of the protein and SNA (0.38 mM) for further haptenation with 21.2 μM AX-B.

### 2.4 Determination of *α*-Enolase Activity

Effects on the enzyme activity were measured in *α*-enolase incubated with vehicle (control), AX or AX-B, using the spectrophotometric method described previously (Baranowski and Wolna 1975). Briefly, the protein (1 μg) was incubated in the absence (control) or presence of 21.2 μM AX or AX-B for 1 h at 37°C. Production of phosphoenolpyruvate was followed for 90 s by the increase in A_220_ of a reaction (1 ml) containing 50 mM Tris/HCl pH 7, 12.5 mM KCl, 10 mM MgCl_2_, 2.5 mM 2-PG, and 21.2 nM of control or AX-treated *α*-enolase. Controls of inhibition reactions contained non-modified *α*-enolase and 100 μM AP-III-a4 (ENOBlock; Selleckchem).

### 2.5 One- and Two-Dimensional Gel Electrophoresis

Analysis of the haptenated proteins by 1D-electrophoresis was carried out on 10% SDS-PAGE gels after denaturation for 5 min at 95°C in Laemmli buffer. Samples loaded on gels contained 1 μg of recombinant protein. Separation of haptenated proteins by 2D-electrophoresis required precipitation to eliminate salts. For this purpose, recombinant proteins (4 μg) were precipitated with 2 vols. of chloroform and 4 vols. of methanol, vortexed for 1 min and centrifuged at 9000 × g for 1 min. The aqueous phase was discarded and 3 vols. of methanol were added, followed by vortexing and centrifugation as above. The supernatant was removed and the pellet dried in a speed-vacuum system at room temperature. Protein pellets were resuspended with isoelectrofocusing buffer (139.2 μl) and separated in 7 cm strips (BioRad) with a linear pH gradient of 3–10. Strips were first rehydrated at 50 V and 50 μA per strip at 20°C for 12 h. After sample loading, isoelectrofocusing was carried out with a program including four steps (1 h each) at: 1) 250 V; 2) 500 V; 3) 1000 V; and 4) 2000 V. The final step was performed at 8,000 V for 2 hours. Strips were then sequentially incubated at room temperature for 15 min in 375 mM Tris/HCl pH 8.8, 6 M urea, 2% (w/v) SDS and 20% (v/v) glycerol (equilibration buffer) containing 130 mM DTT, and later in the equilibration buffer with 135 mM iodoacetamide. Once equilibrated the strips were placed on top of 10% SDS-PAGE gels for the second dimension and later the 2D-gels were subjected to western blotting.

### 2.6 Western Blotting

Proteins from 1D- or 2D-gels were electrotransferred to PVDF membranes (Immobilon-P, Millipore) using a semi-dry system (BioRad) according to manufacturer’s instructions. Membranes were blocked for 1 h with 2% (w/v) low fat milk in TBS containing 0.05% (v/v) Tween-20 (TTBS) before detection of proteins and their modifications using the reagents and conditions listed in Table 1. Antibody and streptavidin dilutions were prepared in TTBS containing 1% (w/v) BSA (Sigma). Signals were developed with ECL chemiluminescence reagents (GE Healthcare) and detected on X-ray films. Quantification was performed with Scion Image software (Scion Corporation).

**TABLE 1:** Conditions and reagents used for detection.

Reagent or primary antibody	Dilution	Secondary antibody	Dilution
	(v/v)^a^		(v/v)
HRP-streptavidin (RPN1231V; GE Healthcare)	1:1,000	—	—
anti-α-enolase (sc-100812; Santa Cruz Biotechnology)	1:500	Rabbit anti-mouse IgG-HRP (P0260; Dako)	1:2,000
anti-penicillin (ref.7220-0004; AbDSerotec)	1:500	Rabbit anti-sheep IgG-HRP (P0163; Dako)	1:2,000
anti-acetyl-lysine (9441; Cell Signaling)	1:5,000	Goat anti-rabbit IgG-HRP (P0448; Dako)	1:2,000

a Incubations with antibodies and HRP-streptavidin lasted generally 1 h and 30 min, respectively.

### 2.7 Mass Spectrometry

Sypro-Ruby (BioRad) stained protein bands from 1D-gels were excised using EXQuest Cutter (BioRad) and washed sequentially with 50 mM ammonium bicarbonate pH 8 and acetonitrile. Trypsinization was carried out with 12.5 ng/μl sequencing grade trypsin (Promega) for 8 h at 37°C in 50 mM ammonium bicarbonate pH 8. Peptide extraction was performed using 100% acetonitrile and 0.5% (v/v) trifluoroacetic acid and the resulting solution dried on a speed-vacuum system. Peptides were resuspended in 6 μl 30% (v/v) acetonitrile and 0.5% (v/v) trifluoroacetic acid and analyzed in an Autoflex III MALDI-TOF/TOF spectrometer (Brucker-Daltonics) at the Servicio de Proteómica y Genómica (CIB) using a linear mode for positive ion detection and retarded extraction and 2,5-dihydroxy-acetophenone (2,5-DHAP) matrix. Results were analyzed using FlexAnalysis software (Brucker-Daltonics) and the SwissProt 2014_03 database. Identification was considered significant (*p* < 0.05) when the score was >59. For analysis of *in vitro* AX-modified *α*-enolase, 2 μl of the sample were diluted with 2 μl of 2% (v/v) trifluoroacetic acid and 2 μl of the matrix solution. Only 1 μl of this mixture was loaded in an 800 μm AnchorChip (Brucker-Daltonics) that was dried at room temperature.

Electrospray ionization mass spectrometry (ESI-MS) was carried out using a LTQ Orbitrap™ XL mass spectrometer (ThermoFisher Scientific) with a linear ionic trap. Three aliquots of recombinant *α*-enolase were taken at time 0 and after 3 h of incubation with AX for desalting through YM-30 Microcon Centrifugal Devices (30 kDa cutoff; Millipore) equilibrated in Milli-Q water by centrifugation at 16,595 g for 6 min. Aliquots were loaded on the equilibrated filter device and centrifuged under the same conditions, after which, processing of the samples followed a different path: 1) one aliquot was washed 4 times with 300 μl Milli-Q water; 2) another was washed twice with 300 μl Milli-Q water and twice with 300 μl of a 1:1 (v/v) ethanol/water solution; and 3) the last aliquot was washed 4 times with 300 μl of a 1:1 (v/v) ethanol/water solution. All washing steps were carried out at 16,595 g until a 50 μl volume was obtained. The recovered protein (96 μg) was diluted with 200 μl water and 200 μl of a 60:40:0.4 (v/v) acetonitrile/water/formic acid solution for their injection into the MS spectrometer at 5 μl/min. Internal calibration was carried out with 20 common plastic contaminants that were detected as a background signal. Results were analyzed with the SEQUEST v1.20 software.

For identification of the modified amino acid(s), the protein was excised from SDS-PAGE gels and digested with sequencing grade trypsin (Promega) and chymotrypsin (Roche) as previously reported (Degani et al., 2017). This protease combination allowed production of peptides of appropriate length, overcoming the lack of effectivity of trypsin on modified lysine residues. The peptide sample was diluted with trifluoroacetic acid 0.1% (v/v) and 5 μl of the peptide solution were injected into a PicoFritTM column (Halo, C18, 2.7 μm 100 Å, 75 μm i. d. ×10 cm, New Objective) connected to an Ultimate 300 RSLCnano System (Dionex) and eluted at 300 nl/min. The chromatography system was connected to a LTQ Orbitrap™ mass spectrometer. Detection of modified peptides and modified residues was performed by means of Proteome Discoverer software (v2.2.0.338, ThermoFisher Scientific), implemented with SEQUEST algorithm designed to match experimental full and tandem mass spectra with theoretical ones obtained from *in silico* digestion of the *α*-enolase sequence (Uniprot P06733). Trypsin and chymotrypsin were selected as cleavage proteases, allowing a maximum of 2 missed cleavages; carbamidomethylation of cysteine was set as a fixed modification (+ 57.02147), whereas oxidation of methionine was allowed as a variable modification, along with a potential mass shift due to adduction of the amoxicilloyl group, mainly on lysine residues. The mass spectrometry proteomics data have been deposited to the ProteomeXchange Consortium via the PRIDE partner repository with the dataset identifier PXD029712.

### 2.8 Molecular Modeling

The *α*-enolase crystal structure (PDB 2PSN) was used and addition of polar hydrogen atoms was performed with AutoDock Tools 1.5.6 (Sanner 1999). The AX structure was obtained from PubChem (code 33613) and its interaction with the protein modeled with AutoDock Vina software (Trott and Olson 2010), considering a 24 Å diameter around K^239^. Nine interaction models were obtained that placed AX close to K^239^, among which those with the most negative binding energy and the shortest distance between the lysine amino and the *ß*-lactam carbonyl groups were considered. PyMOL v1.74 software (Schrödinger, LLC) was used for graphical representation and to measure distances between AX groups and protein residues of interest.

### 2.9 Statistics

A minimum of three independent experiments were carried out and the mean ± SEM calculated. Students *t*-test for paired or non-paired samples was used for the analysis of variables following normal distribution according to the experiment using GraphPad Prism vs6 (GraphPad Software). Differences were considered significant when *p* ≤ 0.05.

## 3 Results

### 3.1 Amoxicillin Haptenation of Human *α*-Enolase

**FIGURE 1:**
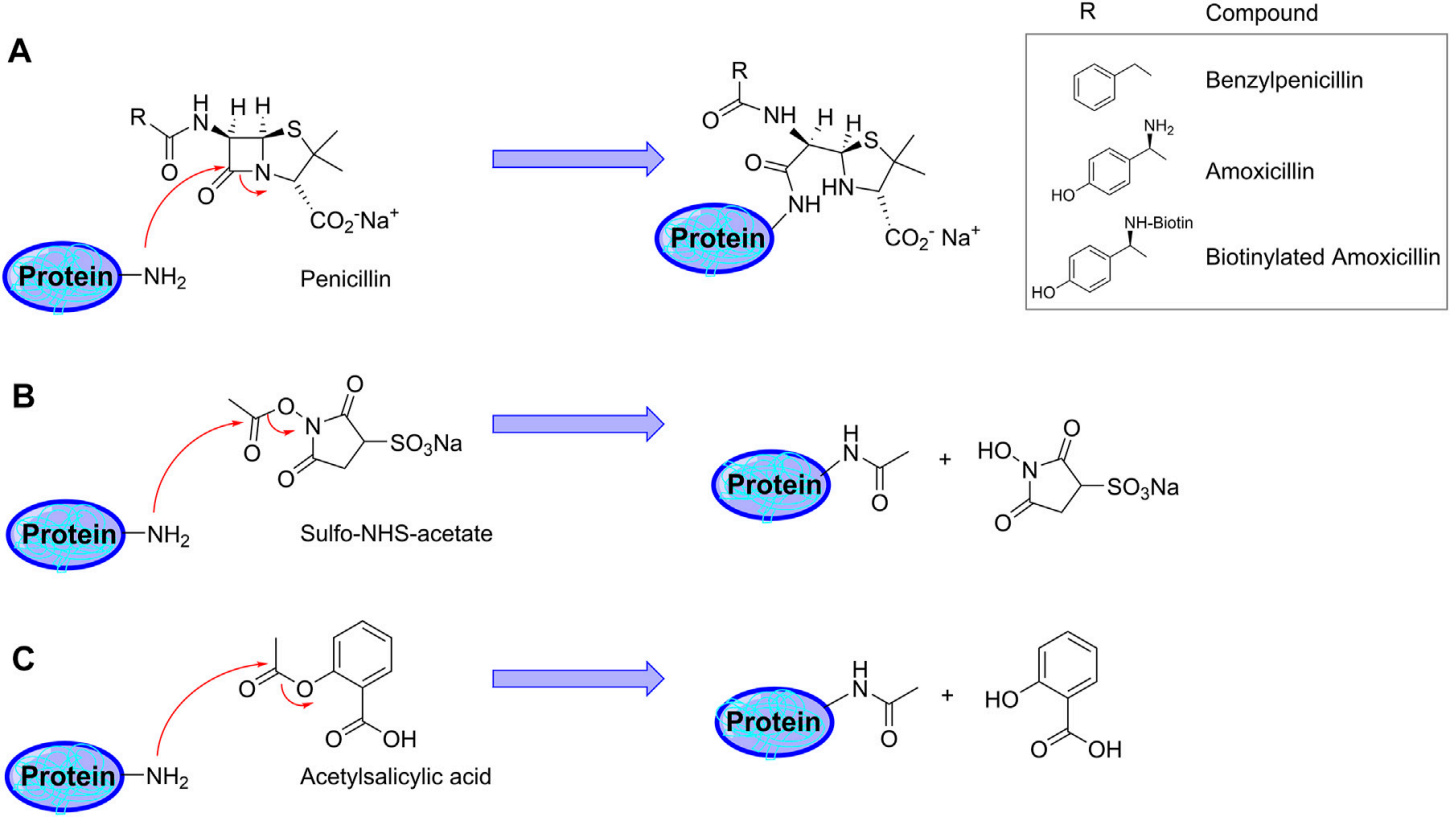
Schematic representation of protein haptenation and acetylation mechanisms by the drugs used in the present study. **(A)** Mechanism of protein haptenation on lysine amino groups by *ß*-lactam antibiotics. The inset shows the structure of “R” in the different antibiotics. **(B)** Acetylation mechanism by sulfo-NHS acetate (SNA). **(C)** Illustration of the acetylation mechanism by acetylsalicylic acid (aspirin).

**FIGURE 2:**
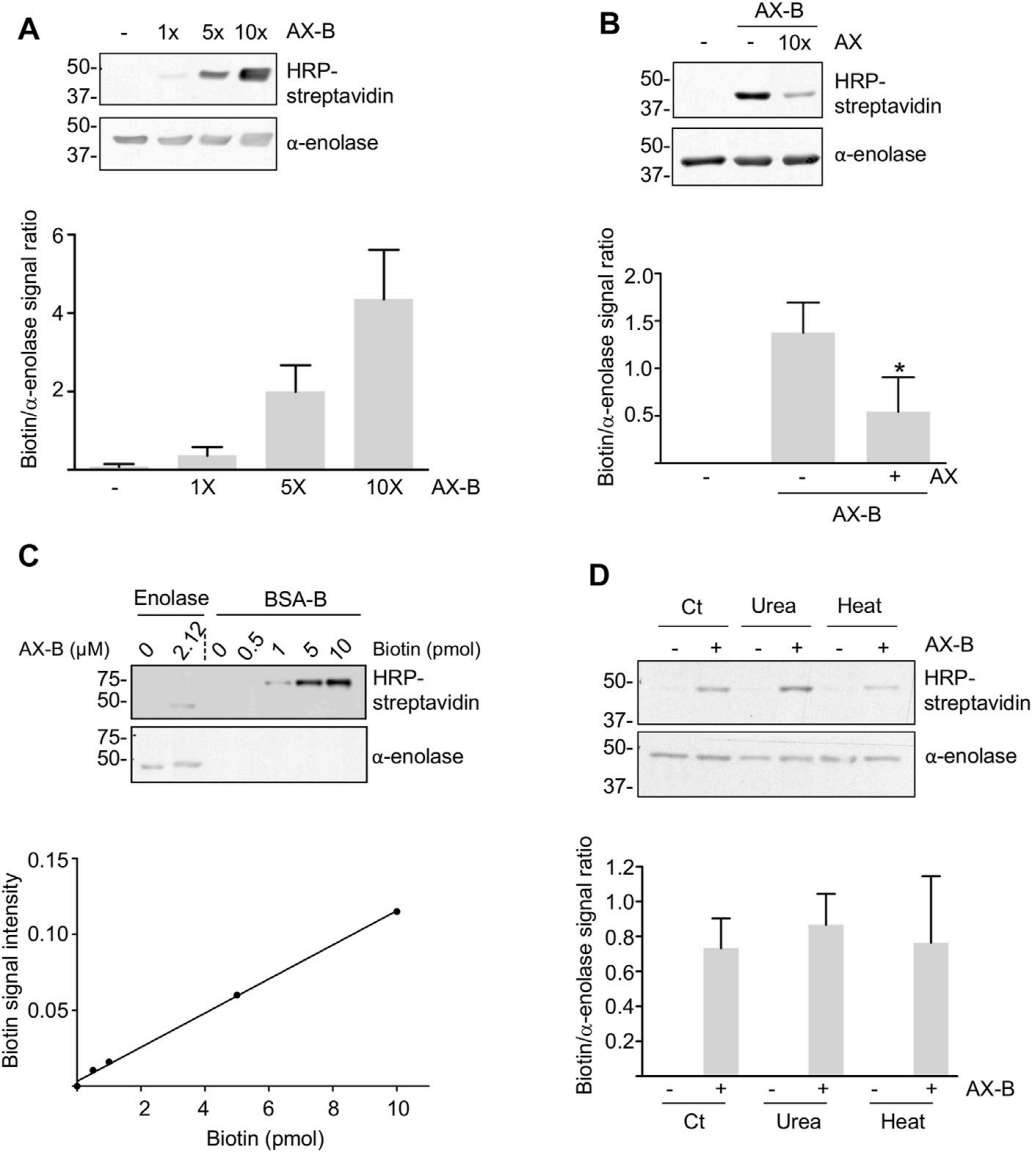
Haptenation of *α*-enolase by amoxicillin. **(A)** Concentration-dependent modification of 2.12 µM *α*-enolase with the indicated molar excess of biotinylated amoxicillin (AX-B) in PBS for 2 h at 37°C detected with HRP-streptavidin. **(B)** Representative western blots of 2.12 µM *α*-enolase modified with a 10-fold molar excess of amoxicillin (AX) for 2 h at 37°C and subsequently incubated with 2.12 µM AX-B. Quantification of the biotin/*α*-enolase signal ratio (mean ± SEM) detected using HRP-streptavidin and anti-α-enolase is shown on the right; *p < 0.05 vs. control incubation without AX. **(C)** Aliquots of biotinylated-BSA (B-BSA) containing the indicated biotin amounts and *α*-enolase incubated with 21.2 µM AX-B in PBS for 1 h at 37°C were separated on SDS-PAGE gels, electrotransferred for biotin detection with HRP-streptavidin and the signal quantified using Scion Image software. The graph below depicts a typical standard curve of B-BSA signal built from Scion Image data and used to estimate AX-B incorporation into *α*-enolase. **(D)** HRP-streptavidin detection of biotin incorporation on control (Ct) or denatured *α*-enolase after incubation for 10 min at 95°C or in the presence of 7 M urea, followed by modification with 10-fold molar excess AX-B (21.2 µM) for 1 h at 37°C. The quantification of the ratio biotin/*α*-enolase signal (mean ± SEM) obtained with HRP-streptavidin and anti-α-enolase is illustrated on the graph.

Amoxicillin is known to modify proteins following the mechanism shown in Figure 1A. Therefore, we incubated recombinant human *α*-enolase with AX-B to induce the formation of AX-protein adducts that were detected with HRP-streptavidin in gel-based assays (Figure 2A). This effect was dependent on the antibiotic concentration. Preincubation of the protein with a 10-fold molar excess of AX before addition of AX-B induced a ∼60% decrease in biotin incorporation into *α*-enolase (Figure 2B), thus suggesting that both AX forms competed for the same binding sites. Quantification of adduct formation using a standard curve of biotinylated-BSA indicated incorporation of 0.7 pmol AX-B, which, assuming a single modification site, corresponds to a 1:30.3 AX-B:*α*-enolase ratio (Figure 2C).

Haptenation may be favored by partial denaturation of the target proteins leading to exposure of sites otherwise hidden in the native tertiary or quaternary structure. Therefore, we explored whether *α*-enolase modification by AX-B was affected by urea or thermal denaturations (Figure 2D). Haptenation levels showed no significant differences between control and denatured *α*-enolase after normalization of the biotin signal with *α*-enolase content (Figure 2D). These results suggested either that no additional modification sites are exposed in the denatured protein or that exposition and modification of new haptenation sites occurred at the expenses of sites that were modified in the native protein.

**FIGURE 3:**
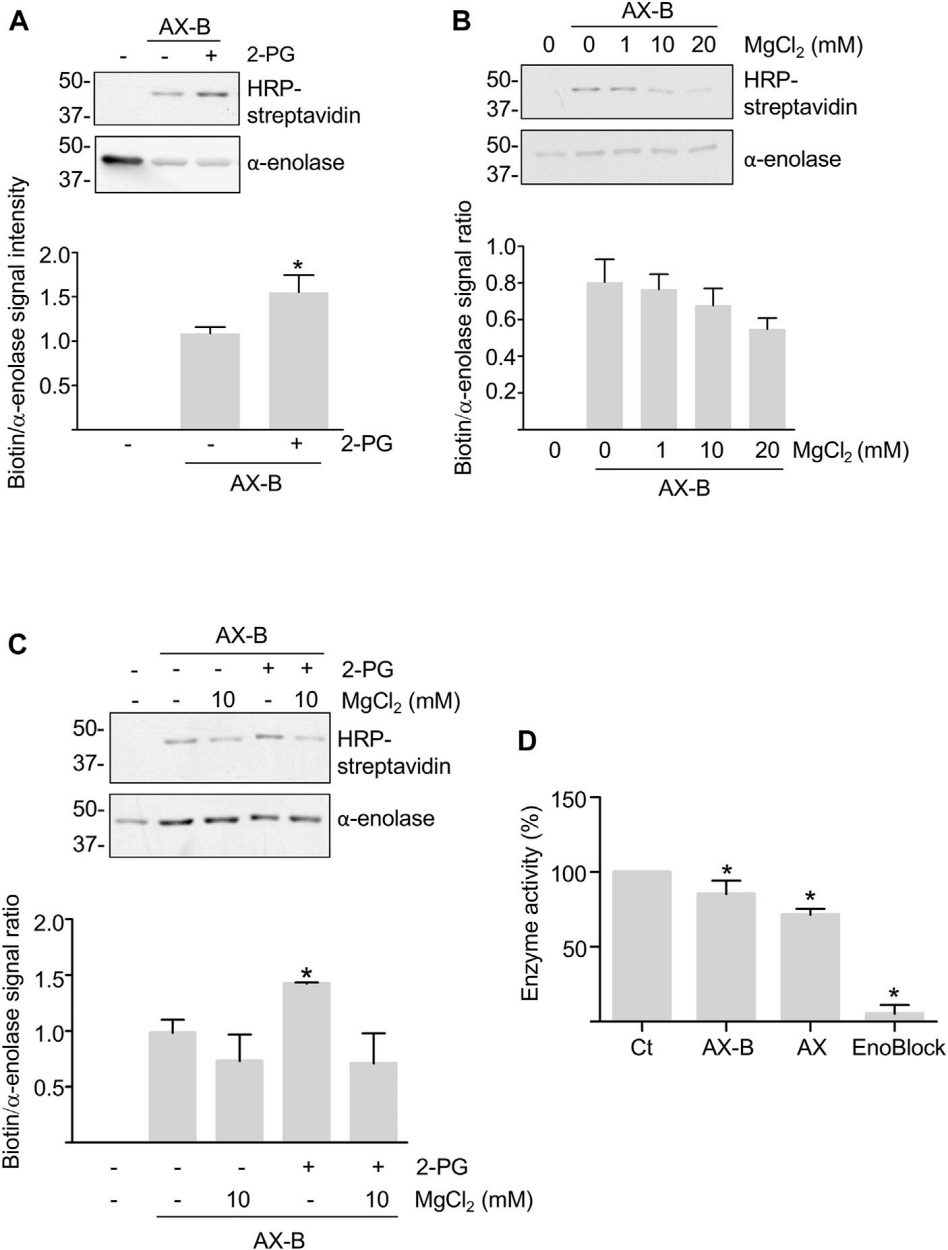
Modulation of haptenation by *α*-enolase substrate and cofactor and effects of haptenation on enzyme activity. **(A)** HRP-streptavidin detection of biotin in equimolar preincubations of *α*-enolase with or without 2.12 mM 2-phosphoglycerate (2-PG) for 1 h at 37°C, followed by modification with 2.12 µM biotinylated amoxicillin (AX-B) for an additional hour at the same temperature. **(B)** HRP-streptavidin detection of biotin incorporation on *α*-enolase preincubated with the indicated MgCl_2_ concentrations for 1 h at 37°C, followed by modification with AX-B as in **(A)**. **(C)** Haptenation with AX-B 21.2 µM for 1 h at 37°C of *α*-enolase at 2.12 µM preincubated for 1 h at the same temperature in the presence or absence of 2.12 mM 2-PG and/or 10 mM MgCl_2_ followed by western blotting using HRP-streptavidin and anti-α-enolase. Graphs below panels A, B, and C show quantifications (mean ± SEM) of the biotin/*α*-enolase signal ratio obtained in at least three independent experiments; *p < 0.05 vs. AX-B incubations in the absence of substrate and/or cofactor. **(D)** Activities (mean ± SEM) of control (Ct) and 21.2 µM amoxicillin (AX) or AX-B treated *α*-enolase for 1 h at 37°C obtained in five independent experiments. ENOBlock (100 µM) inhibition of *α*-enolase activity measured under the same conditions is also shown. *p < 0.05 vs. control.

While haptenation may induce structural changes in the protein or partially block access of the substrates and cofactors to the active site, both substrates and cofactors may also modulate adduct formation. Hence, modification by AX-B was analyzed in the presence of MgCl_2_ and/or 2-PG concentrations typically used for *in vitro* activity assays. Millimolar concentrations of 2-PG induced a 50% increase in adduct formation (Figure 3A). However, a trend toward decreased adduct formation was found in the presence of MgCl_2_ that was concentration-dependent (Figure 3B). Combination of 2-PG and MgCl_2_ precluded the increased haptenation induced in the presence of the substrate alone (Figure 3C). Furthermore, the possibility that haptenation, as other protein PTMs, controls *α*-enolase activity was also explored. For this purpose, *α*-enolase activity was measured in control reactions, after incubation with AX or AX-B and in the presence of the ENOBlock inhibitor (Figure 3D). Compared with control activity levels, slight, but significant, decreases (10–15%) in phosphoenolpyruvate production in the presence of AX or AX-B were detected, together with a near abolishment of *α*-enolase activity in the presence of the inhibitor (Figure 3D).

### 3.2 Analysis of Haptenated *α*-Enolase Forms

**FIGURE 4:**
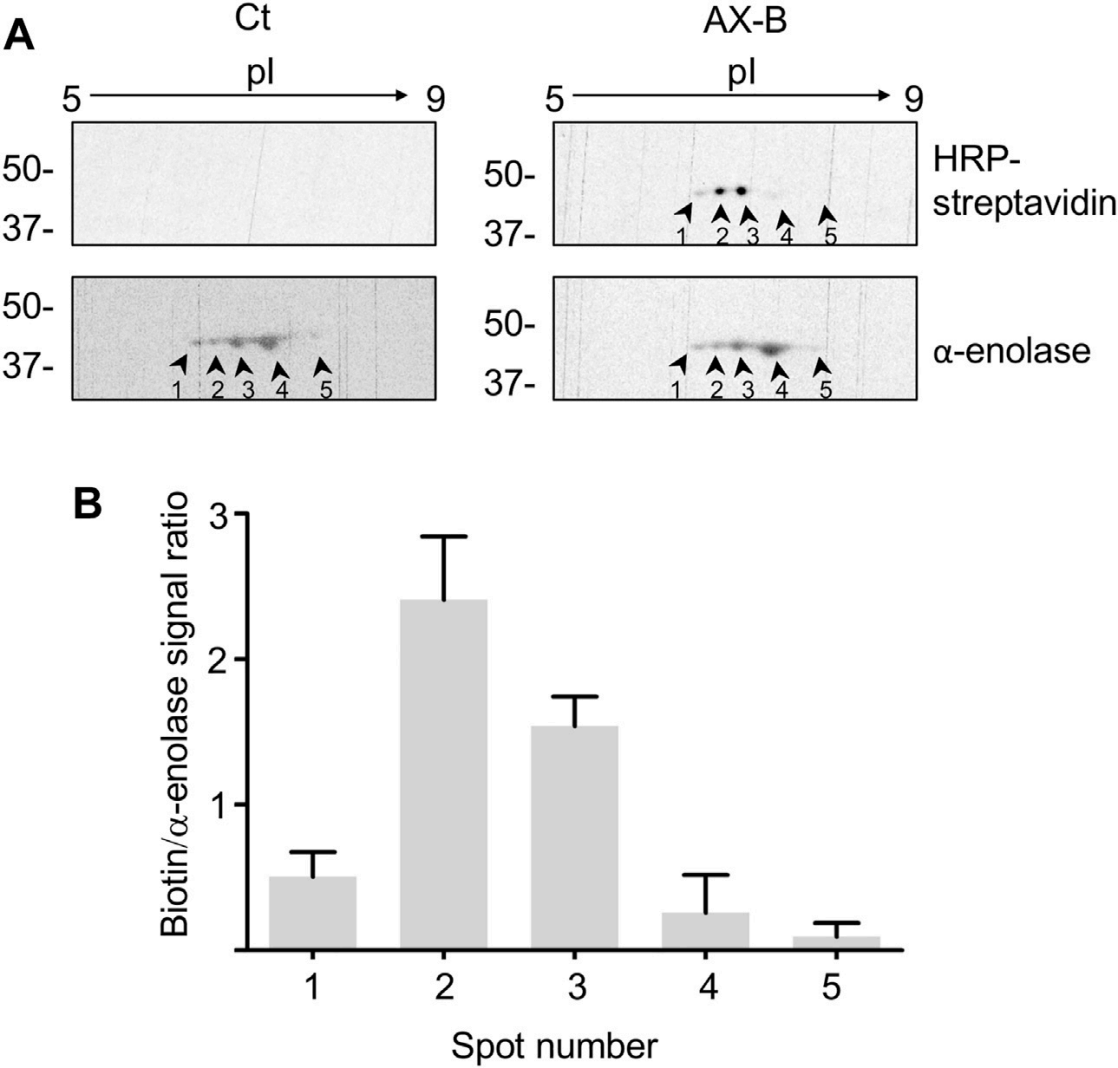
Amoxicillin haptenation of different α-enolase forms. **(A)** Separation by 2D-electrophoresis of *α*-enolase incubated in the absence (control, Ct) or presence of 21.2 µM biotinylated amoxicillin (AX-B) for 1 h at 37°C. HRP-streptavidin followed by anti-*α*-enolase incubation were used to detect the modification and the protein, respectively; five positive spots were found and labeled 1–5. **(B)** Quantification (mean ± SEM) of the ratio biotin/α-enolase signal for each spot from three independent experiments.

Haptenated *α*-enolase was detected previously in lysates as well as in the conditioned medium of RPMI 8866 lymphocytes treated with AX or AX-B and in this setting several spots of the enzyme were observed (Sánchez-Gómez et al., 2017). The diverse isoelectric points (pIs) exhibited by these spots suggest the existence of several *α*-enolase proteoforms containing diverse PTMs. Differences in susceptibility to AX-haptenation may arise from the presence of this variety of PTMs on *α*-enolase and a putative interplay between these modifications. Therefore, it was of interest to ascertain the presence of proteoforms in the recombinant preparation of *α*-enolase used in the current study. In order to explore this aspect, native and AX-B-modified recombinant *α*-enolase were analyzed by 2D-electrophoresis (Figure 4A). In both cases, a similar *α*-enolase pattern including 5 spots was found, likely representing different proteoforms arising during bacterial expression. However, the biotin signal indicated higher haptenation levels in proteoforms with a more acidic pI (spots 1, 2 and 3). In fact, spots 2 and 3 were the most intensely labeled with AX-B, showing 5- and 3-fold higher biotin levels than spot 1 (Figure 4B). Given the fact that haptenation by *ß*-lactam antibiotics usually occurs on lysine residues, the possibility exists that AX-B haptenation could influence the pI of *α*-enolase with the haptenated protein appearing at a more acidic pI.

### 3.3 Interplay Between Acetylation and Haptenation of *α*-Enolase

**FIGURE 5:**
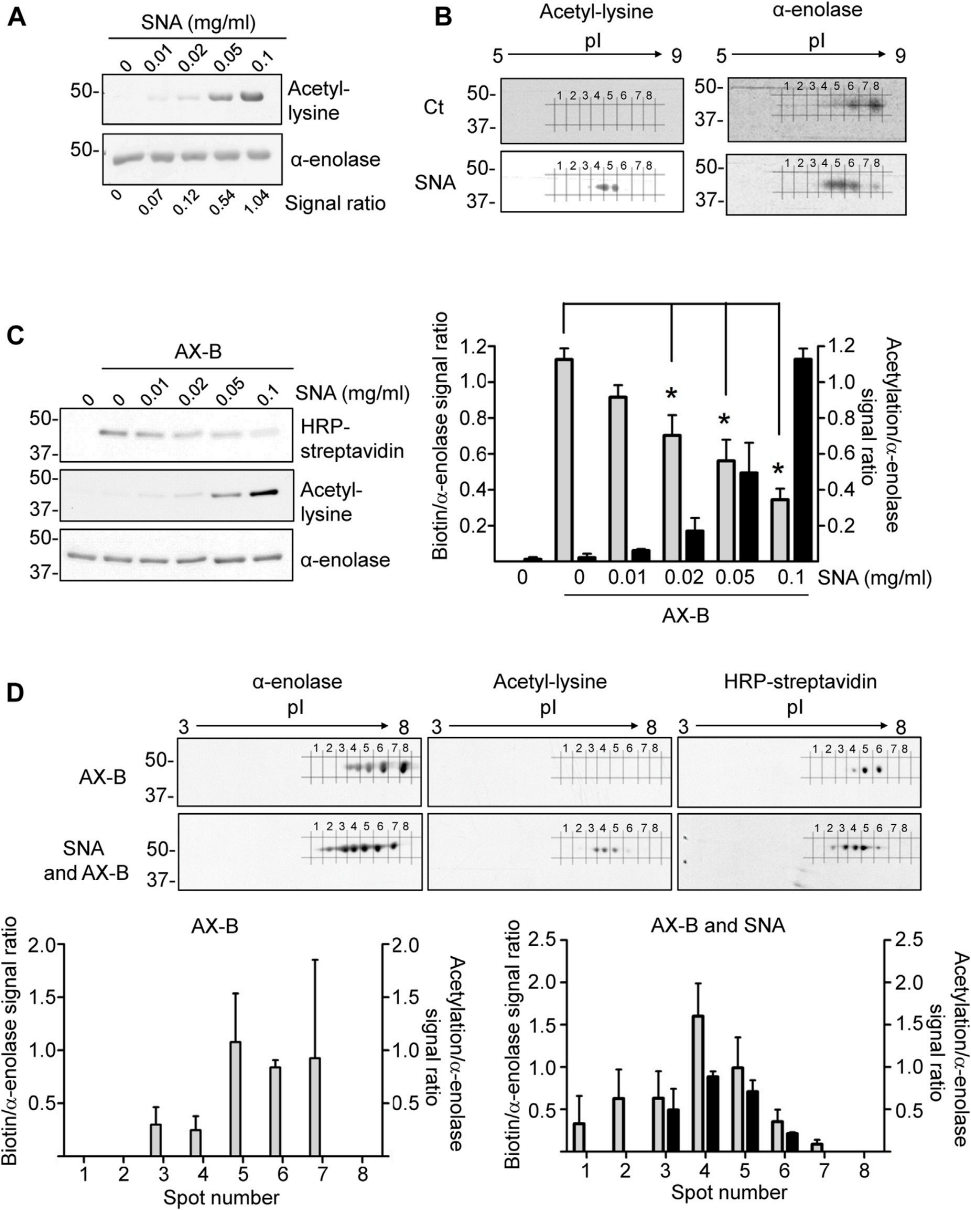
Interplay between acetylation and amoxicillin haptenation of α-enolase. **(A)** Acetylation of *α*-enolase (2.12 µM) with different concentrations of sulfo-NHS-acetate (SNA) representing 18-, 36-, 90-, and 120-fold molar excess for 1 h at RT evaluated by immunoblotting with anti-acetyl-lysine and anti-*α*-enolase. Quantification performed with Scion Image software of the ratio acetyl-lysine/*α*-enolase from a typical experiment is shown below. **(B)** Representative images of three independent experiments of *α*-enolase modification with a 180-fold molar excess of SNA for 1 h at RT, separated by 2D-electrophoresis and analyzed by immunoblotting as in **(A)**. **(C)** Haptenation of *α*-enolase with different acetylation levels, obtained by preincubation with 18-, 36-, 90-, and 180-fold molar excess SNA for 1 h at RT, was achieved by subsequent addition of biotinylated amoxicillin (AX-B; 21.2 µM) for 1 h at 37°C. Detection was carried out sequentially with HRP-streptavidin, anti-acetyl-lysine, and anti-*α*-enolase, performing a stripping step of the blots between the different detection steps; representative images of a typical experiment are shown. The graph depicts quantification (mean ± SEM) of the ratios biotin/α-enolase (grey bars) and acetyl-lysine/*α*-enolase (black bars) from three independent experiments; *p < 0.05 vs. control with AX-B. **(D)** Separation of proteoforms by 2D-electrophoresis from control and acetylated *α*-enolase after their haptenation with AX-B (21.2 µM) for 1 h at 37°C; acetylation was carried out with SNA (0.38 mM) for 1 h at RT. Detection was performed by incubation with HRP-streptavidin, anti-acetyl-lysine, and anti-*α*-enolase; representative images of a typical experiment are shown. Lower panels show quantification of the ratios biotin/*α*-enolase (grey bars) and acetyl-lysine/*α*-enolase (black bars) of the corresponding spots numbered 1 to 8 in the blots. Results are shown as mean ± SEM from three independent 2D-experiments; *p < 0.05 vs. control with AX-B.

Acetylation is among the PTMs identified in *α*-enolase in several studies. This modification occurs frequently on lysine residues (Figure 1B), which are also a preferred target for haptenation by AX. Therefore, we next studied the impact of *α*-enolase acetylation on its AX modification. For this purpose, incubations with sulfo-NHS-acetate (SNA), which reacts with primary amines in proteins, were carried out. Acetylated *α*-enolase levels were then evaluated using 1D- and 2D-electrophoresis followed by immunoblotting with anti-acetyl-lysine and anti-*α*-enolase. Separation on 1D-gels showed a concentration-dependent increase in *α*-enolase acetylation, as well as apparent lack of basal modification of the recombinant protein (Figure 5A). Additionally, 2D-electrophoresis revealed a change in the pattern of proteoforms between control and acetylated *α*-enolase (Figure 5B), acetylation leading to an increase in the abundance of those spots exhibiting lower pIs. Moreover, these were the proteoforms showing a stronger acetylation signal, as expected from the putative decrease in the protein positive charge induced by this PTM.

Next, the impact of acetylation on AX-B modification was explored using increasing SNA concentrations to obtain *α*-enolase proteoforms with different acetylation levels, which were subjected to AX-B haptenation. Immunoblots of 1D-gels showed a concentration-dependent increase in *α*-enolase acetylation, together with decreased AX-B haptenation, as indicated by the corresponding biotin signal (Figure 5C). This inverse correlation was significant at SNA concentrations over 20 μg/ml (36-fold molar excess). Further analysis of the susceptibility of acetylated *α*-enolase to haptenation by 2D-electrophoresis indicated higher modification levels at proteoforms exhibiting lower pIs (spots 2–6) than those modified in control *α*-enolase (spots 3–6) (Figure 5D). Moreover, stronger haptenation signals corresponded to spots 3, 4, and 5 in SNA-treated *α*-enolase vs. spots 5 and 6 in the non-acetylated protein.

### 3.4 Haptenation and Acetylation of *α*-Enolase by Clinically Used Drugs

**FIGURE 6:**
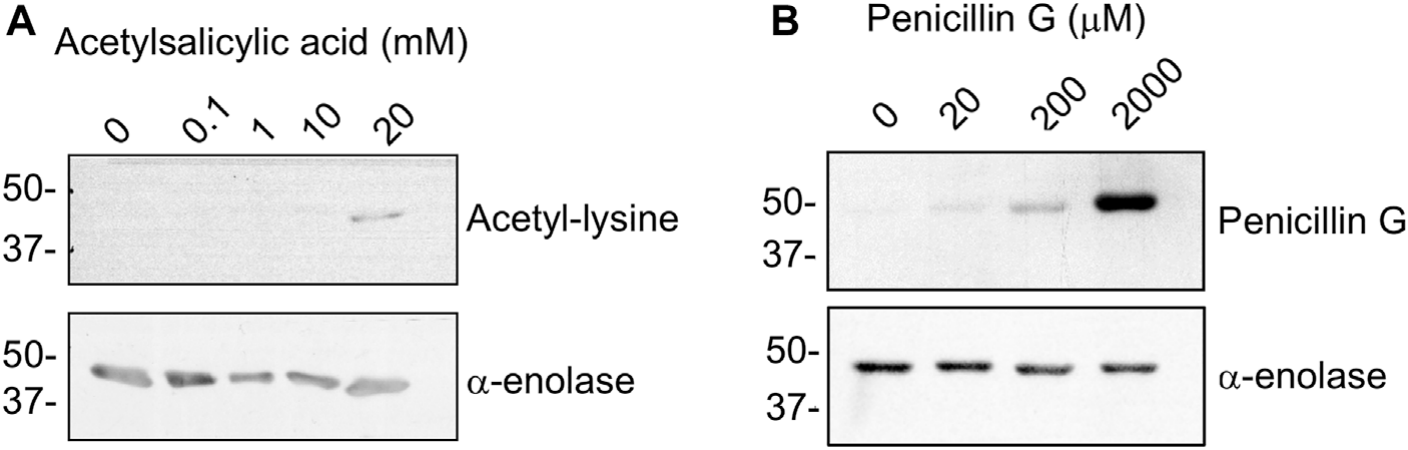
Acetylation and haptenation of α-enolase produced by other agents. **(A)** *α*-enolase (2.12 µM) acetylation induced by the indicated concentrations of acetylsalicylic acid for 1 h at 37°C detected with anti-acetyl-lysine and anti-*α*-enolase. **(B)** Haptenation of *α*-enolase (2.12 µM) with the indicated concentrations of penicillin G for 1 h at 37°C detected with anti-penicillin and anti-*α*-enolase. Images shown are representative blots from three and two assays, respectively.

Drugs commonly used in the clinic, such as acetylsalicylic acid, can produce non-enzymatic acetylation of proteins (Figure 1C). Hence, we explored the possibility of *α*-enolase acetylation using a range of aspirin concentrations followed by immunoblot detection of the modification with anti-acetyl-lysine (Figure 6A). *In vitro* lysine acetylation on *α*-enolase was only observed at high concentrations of the drug (20 mM), which are well-above those found in plasma during adverse reactions (0.8–1.6 mM). Thus, although both SNA and aspirin were able to produce *α*-enolase acetylation, the latter seemed less effective.

Additionally, there is no information to date regarding whether *α*-enolase haptenation by AX can be extended to other *ß*-lactam antibiotics. Therefore, the recombinant protein was incubated with a range of penicillin G concentrations and haptenation detected using an anti-penicillin antibody (Figure 6B). The results obtained indicated a concentration-dependent incorporation of penicillin into *α*-enolase. However, comparison between penicillin G and AX haptenation data was precluded by the diversity of detection methods used and, in turn, putative differences in haptenation efficiency by both antibiotics could not be established at this point.

### 3.5 Identification of the Amoxicillin Haptenation Site on *α*-Enolase

**FIGURE 7:**
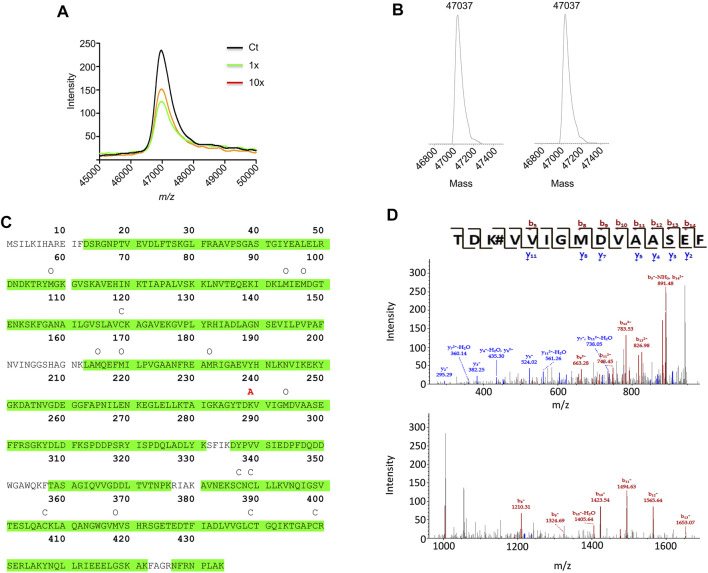
Mass spectrometry analysis of amoxicillin haptenated α-enolase. **(A)** MALDI-TOF MS spectra of control *α*-enolase (2.12 µM) and incubations with equimolar or 10-fold molar excess amoxicillin (AX) for 1 h at 37°C. **(B)** Deconvoluted ESI-MS spectra of *α*-enolase (2.12 µM) incubations with 10-fold molar excess AX at 37°C for 0 **(left)** and 3 h **(right)**. **(C)** Human *α*-enolase sequence highlighting in green the peptide sequences identified by LC-MS/MS after trypsin/chymotrypsin digestion. Symbols above the sequence indicate modifications detected by this method as follows: C, carbamidomethylation; O, oxidation; and A, AX haptenation. **(D)** Spectra of the haptenated peptide collected in the collision induced dissociation mode in the 150–950 m/z (upper spectrum) and 950–1,650 m/z ranges (lower spectrum); b- and y-series are shown on the top.

AX-haptenation increases the mass of the target protein and this change can be determined in top-down analysis by mass spectrometry. Therefore, *α*-enolase after modification with AX was analyzed by MALDI-TOF (Figure 7A). The spectra recorded showed a single peak of 46,972.7 m/z in all the samples (control and AX-modified), as expected for the unmodified protein. ESI-MS deconvoluted spectra of control samples (time 0 of AX incubation) showed a 47,037 Da peak for *α*-enolase, which was also the single peak observed in spectra of samples incubated for 3 h with AX (Figure 7B). We noted a small difference in m/z between the two approaches, likely due to different experimental settings. Lack of detection of haptenated *α*-enolase by these approaches may rely on two facts: 1) the presence of a small proportion of AX-haptenated protein under our conditions; or 2) a decrease in the ionization of the modified protein. To overcome these difficulties, a bottom-up approach was chosen to detect the haptenated peptides in samples modified for 16 h using a 1:1,000 protein:AX ratio and a LTQ-Orbitrap XL spectrometer. Identification of the haptenated peptides was based on addition of 365 Da to nucleophilic residues such as histidine, cysteine and mainly lysine. Protein digestion with a trypsin/chymotrypsin combination provided 89.4% coverage of the *α*-enolase sequence, and hence included most putatively haptenated lysine residues (Fig 7C). Out of all these peptides, an increase of mass compatible with AX incorporation was detected in the ^237^TDKVVIGMDVAASEF^251^ fragment. Not all the ions of the b- and y-series were detected in a detailed analysis of the peptide fragmentation spectra. In fact, only b5 and b8-b14 ions were identified, all of them containing K^239^ and exhibiting a 365 Da mass increase. Additionally, no mass increment was found in the y2-y5, y7, y8 and y11 ions, none of them containing K^239^ (Figure 7D). Overall, these results indicated that AX incorporation occurred on one of the first four residues of the 237–251 peptide, most probably on K^239^. Moreover, this approach also detected the existence of additional modifications in *α*-enolase (Fig 7C), such as carbamidomethylations (C^427^, C^429^, C^447^, C^479^ and C^499^) and oxidations (K^54^, M^94^, M^97^, M^255^, M^259^, M^272^, M^334^, M^458^, some of which may be produced during sample processing.

### 3.6 Molecular Docking of Amoxicillin on the Human *α*-Enolase Structure

**FIGURE 8:**
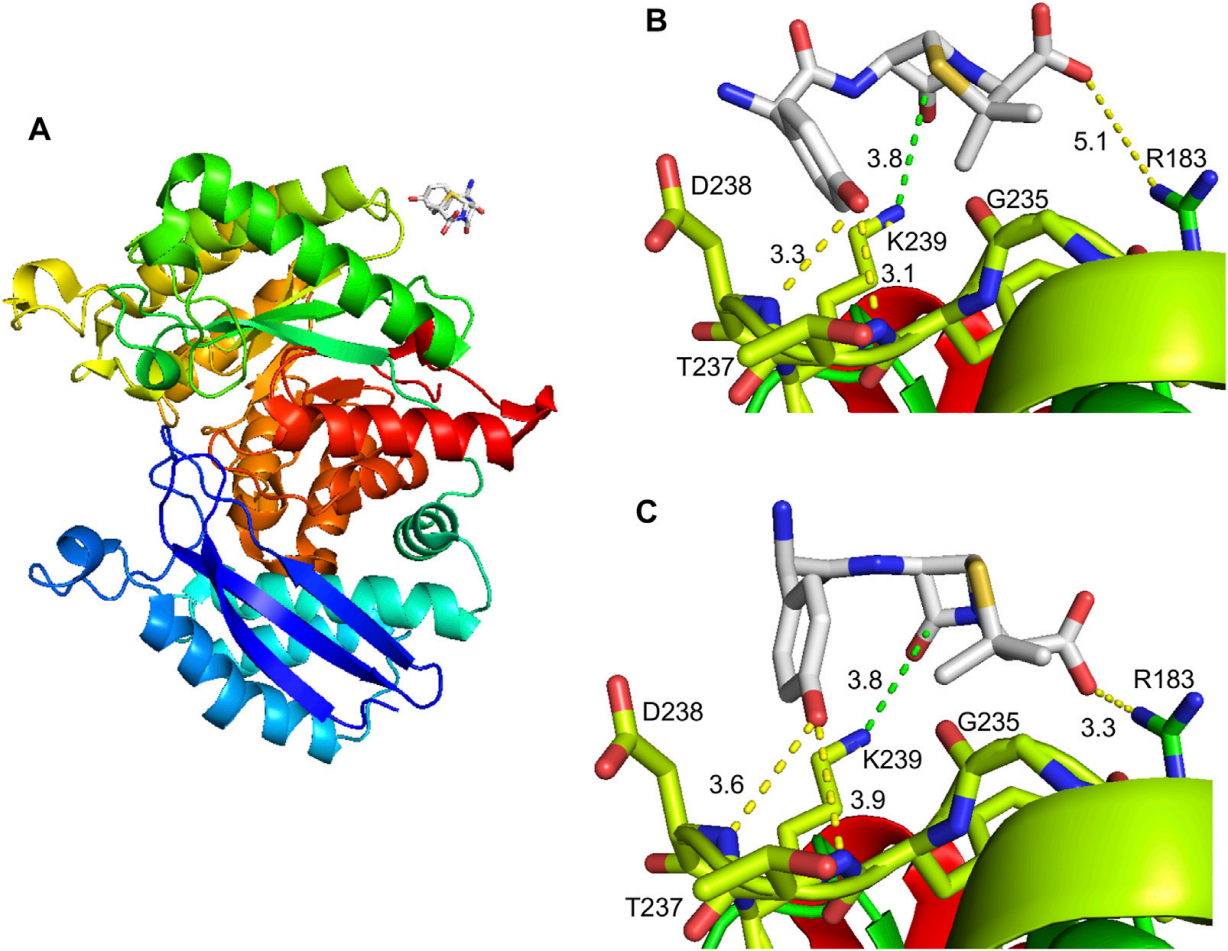
Molecular modeling of the amoxicillin modification on α-enolase. **(A)** General view of the *α*-enolase (PDB 2PSN, chain A) interactions with amoxicillin (AX); the color gradient indicates the direction of the sequence towards the N-terminal (blue) or C-terminal (red) ends. **(B)** Structure in which the *ß*-lactam ring is placed close to K^239^ with an affinity energy of −3.7 kcal/mol. **(C)** Structure in which the *ß*-lactam ring is placed close to K^239^ with an affinity energy of −3.6 kcal/mol. Discontinuous lines in **(B)** and **(C)** show distances between the *ß*-lactam carbonyl group of AX and K^239^ amino group (green) or between other AX groups and protein residues.

Information regarding AX interaction with its target residue could help in the identification of consensus haptenation sites, as well as in the design of compounds that could interfere with this process, in turn, precluding adverse drug reactions. For this purpose, putative interactions of *α*-enolase with AX were explored in a docking space of 24 Å around K^239^ using molecular modeling with Autodock Vina. This approach identified a single AX binding site in this area located at the surface of human *α*-enolase structure (2PSN), in a stretch comprising the central part of the sequence (Figure 8A). Docking of AX into the crystal structure provided nine energy favored binding modes (−3.7 to −3.0 kcal/mol), out of which two placed the carbonyl group of the *ß*-lactam ring close to the K^239^ amino group, putatively favoring nucleophilic attack for adduct formation (Figures 8B,C). Binding energies were similar in both models, −3.7 and −3.6 kcal/mol, and AX binding could be stabilized by interactions involving other residues. Precisely, the AX carboxyl group and the R^183^ lateral chain, as well as the hydroxyl of the AX phenol group and T^237^ and the D^238^ backbone.

## Discussion

Haptenation of proteins by AX can determine their recognition by the immune system as foreign antigens and the trigger of an allergic response mediated by IgE that occurs within 1 h of administration (Antúnez et al., 2006; Torres et al., 2011). Therefore, identification of AX target proteins, their modification sites and the mechanisms of their haptenation has become an important line of research to design strategies for preventing or counteracting these unwanted effects. The increasing prescription of AX, alone or in combination with clavulanic acid, has also raised the incidence of allergic reactions. In fact, besides the already known responses against AX, allergic reactions against clavulanic acid are being identified (Torres et al., 2010).

From the chemical point of view, the reactivity of penicillins, underlying haptenation reactions, is related to the high tension within the *ß*-lactam ring due to the chemical structure resulting from the condensation of the *ß*-lactam with the thiazolidine ring. This increases the ability of the carbonyl group of the *ß*-lactam to react with nucleophiles. In this context, several amino acid residues could act as nucleophiles, mainly, the amino-terminal groups, the amino groups of the side chains of lysine residues, the imidazole amino group of histidine residues or the thiol group of cysteine residues (Ariza et al., 2012). All these nucleophiles are able to react with AX via *ß*-lactam ring opening, which involves the formation of different bonds depending on the nucleophilic group involved. When amino nucleophiles attack AX, a conjugate is formed via a stable and irreversible amide bond (Figure 1A). Among the nucleophilic sites present in human serum albumin, lysine residues are the most reactive sites towards AX (Ariza et al., 2012). On the other hand, thiol nucleophiles have been reported to react with penicillins giving rise to a thioester intermediate, which is reactive and hydrolyses in the case of benzylpenicillin, or induces a cyclisation of AX with the formation of AX-diketopiperazine (Llinás et al., 2000; Pajares et al., 2020). Due to the lower nucleophilicity of the serine hydroxyl group, its *in vitro* conjugation with AX is not expected. Nevertheless, the interaction between the carbonyl group of the *ß*-lactam ring and the hydroxyl group of serine or the thiol group of cysteine at the active center of transpeptidase enzymes (involved in bacterial peptidoglycan biosynthesis), which results in an inactive acylated form of the enzyme, is well known (Ariza et al., 2012; Lohans et al., 2019).

Regarding protein targets, albumin was the main target of AX-modification in serum (Ariza et al., 2012), and K^190^ the main haptenation site identified (Garzon et al., 2014). Other AX haptenation targets, such as *α*-enolase, were modified not only in serum (Ariza et al., 2012), but also intracellularly and in extracellular vesicles (Sánchez-Gómez et al., 2017) paving the way for their transport to other cells to expand their adverse effects. As described for albumin (Ariza et al., 2014), *α*-enolase can be modified by AX-B *in vitro* in a concentration-dependent manner and at the same site haptenated by AX as suggested from decreased incorporation of AX-B on previously AX-modified protein. AX preferentially haptenates lysine residues. Among the 38 lysine residues present in the *α*-enolase sequence, 33 appear exposed in the 2PSN structure. Remarkably, only K^239^ was found haptenated under our experimental conditions, thus indicating a certain selectivity of the modification, although we cannot exclude that other residues not covered by the LC-MS/MS, as depicted in Figure 7C, can be modified as well. The calculated biotin/α-enolase ratio suggests modification of a 3.3% of the protein at a single site, K^239^. Such a low percentage of modification can be ascribed to several facts affecting both the protein and the drug. Regarding the protein, putative low reactivity of the target residue, due to poor exposition, or the presence of nearby groups altering its properties, can influence the extent of haptenation achieved. Conversely, although the *ß*-lactam reactivity of AX-B is equivalent to that of AX, the biotin linked to the AX amino group could provide steric interactions that interfere with haptenation and putative decomposition of AX-B during the incubation can also take place. In fact, factors such as a thiol reducing agents (e.g., GSH) can contribute to AX conversion into its diketopiperazine, in turn, decreasing protein haptenation (Pajares et al., 2020). However, the conversion extent achieved will largely depend on the concentration of such reagents in the plasma or intracellularly, which is nearly 1000-fold higher in the latter.

Susceptibility to haptenation can be influenced by the presence of additional PTMs on *α*-enolase, whether they have the same target residue or just induce local changes in its environment. The number of PTMs that can be found in a protein is huge and their influence can be so diverse that the outcome is difficult to predict. Immunodetection of several spots with identical size but different pI can be an indicator of the presence of diverse PTMs or different levels of the same PTM on a protein. This is the case of *α*-enolases obtained from different organisms (Foth et al., 2008), tissues or pathologies (Ochi et al., 2002; Tomaino et al., 2011; Peng et al., 2013). In fact, studies on bacteria or human cells have identified phosphorylation, acetylation, or methylation among PTMs that could cause pI changes that explain this *α*-enolase pattern (Zhou et al., 2010). An example of these effects on mobility is provided by serine/threonine kinase PrKc phosphorylation of *B*. *anthracis* enolase that occurs mainly on three residues of its C-terminal end, leading to the apparition of several spots with different pIs upon 2D-electrophoresis (Virmani et al., 2019). The present *in vitro* study adds to this list of PTMs the identification of several carbamidomethylations and oxidations on the molecule, which occur during the haptenation reaction and that, together with PTMs already carried by the isolated *α*-enolase used, can account for the diversity of *α*-enolase spots detected by 2D-electrophoresis even in control reactions without AX.

Changes in *α*-enolase PTM levels can be the result of the protein exposure to different contexts, as in the case of *α*-enolase from peripheral blood mononuclear cells of rheumatoid arthritis patients (Arito et al., 2015), a disease in which the protein behaves as an autoantigen (Klareskog et al., 2008; Wegner et al., 2010). Cells of these patients exhibit increased *α*-enolase acetylation levels compared to those of healthy controls, although these enhanced acetylation levels do not change the protein’s antigenicity (Arito et al., 2015). Additionally, the same PTM may lead to opposite effects due to its introduction on diverse residues or by different enzymatic or non-enzymatic mechanisms. This is also the case of *α*-enolase acetylation, whose increased levels associate with enhanced protein activity in rheumatoid arthritis samples (Arito et al., 2015), while suppressing the enzyme activity upon acetylation (Nakayasu et al., 2017) or phosphorylation (Virmani et al., 2019) in bacteria, as well as in HEK293 samples incubated with millimolar concentrations of salicylic acid, the main metabolite of aspirin (Choi et al., 2019). None of these seem to be the effect exerted by AX on *α*-enolase activity, which only shows a slight decrease in phosphoenolpyruvate production. Such an outcome may be explained by the modest AX-haptenation levels attained, but could also result from opposite or combined effects of AX-modification and putative PTMs carried by the recombinant *α*-enolase used in our haptenation assays.

An interplay between PTMs inducing charge changes in the molecule and haptenation was also suggested by the higher haptenation levels found on low pI spots after *α*-enolase acetylation with SNA. However, whether haptenation on lower pI forms correlates with a preferential modification of certain acetylated *α*-enolase forms or to a change of charge induced by haptenation itself remains unclear. Moreover, some PTMs may share the same type of target residue than AX, and hence preclude or decrease haptenation. This last behavior is what we observe upon *in vitro* acetylation, a PTM that removes the positive lysine charge (Christensen et al., 2019), and that occurs frequently in glycolytic enzymes (Nakayasu et al., 2017). Nevertheless, the fact that haptenation takes place on acetylated *α*-enolase proteoforms of low pI suggests either that K^239^ may not be the preferential lysine targeted by SNA and, on the other hand, that a certain degree of acetylation on other sites may favor exposure or reactivity of this residue towards AX-B. These two options are also supported by the identification of K^120^, K^126^, and K^256^ as acetylation sites on *α*-enolase of healthy peripheral blood mononuclear cells (Arito et al., 2015).

According to the crystal structure (Kang et al., 2008), K^239^ locates at the protein surface, its side chain facing the external shell of each subunit near the monomer-monomer interface (Figure 8). This lysine is surrounded by E^180^, R^183^, D^238^, and Y^236^, whose lateral chains also point toward the protein surface at less than 10 Å of the NZ atom of K^239^. Among these residues, R^183^ lies at the monomer-monomer interface, whereas the OE2 atom of E^180^ is placed at 2.9 Å of the NZ atom of K^239^, a distance allowing ionic bonding (Kumar and Nussinov 2002). AX-haptenation of K^239^ imposes an increase in the lateral chain size of this residue that needs to be accommodated by the structure, putatively by small local changes that can be transmitted to nearby residues such as R^183^, and hence to the monomer-monomer interface. As *α*-enolase dimerization is required for enzyme activity (reviewed in (Pancholi 2001)), we can therefore propose that the small activity decrease detected in the presence of AX may derive from a slight perturbation at the monomer-monomer interface induced directly by AX-haptenation or indirectly by AX binding to the protein surface. Another possibility to explain effects on activity relies in the position of K^239^ itself, on a loop at the bottom of the (*αβ*)_8_ barrel. Although, this location places K^239^ opposite to the loops constituting the active site of the C-terminal domain (Kang et al., 2008), this lysine lays two and six residues apart from the start of a *ß*-strand from the inner face of the barrel and from D^245^ involved in Mg^2+^ binding, respectively. Therefore, perturbations in the K^239^ environment by the AX moiety may be transmitted and influence indirectly enzyme activity. Additionally, ionic bonding between K^239^ and E^180^ would be precluded by haptenation, further contributing to destabilization of the enzyme structure at the bottom of the barrel.

The crystal structure also shows that the lateral chain of K^239^ is exposed at the protein surface (Kang et al., 2008), and hence rules out the possibility that poor AX accessibility may be responsible for the low haptenation observed. In fact, the large conformational changes induced by thermal and urea denaturation that are expected to unfold the native structure do not increase *α*-enolase haptenation levels. In contrast, smaller structural alterations due to substrate and cofactor binding to the active site and catalysis influence haptenation. Increased modification in the presence of 2-PG could be explained by local structural alterations produced by unspecific binding of the substrate to other areas of the protein in a context (absence of Mg^2+^) where conformational changes allowing its binding to the active site are precluded (Faller et al., 1977). Conversely, when Mg^2+^ is present, conformational changes in the active site occur allowing 2-PG binding (Faller et al., 1977), and hence precluding additional haptenation. These changes and where they take place remains to be studied.

Finally, we should highlight that K^239^ is a highly conserved residue among enolases, including neuronal ENO2. Nevertheless, this lysine is substituted by glutamine in several of the allergenic enolases included in the AllFam database. Moreover, K^239^ is not included in any of the peptides or epitopes of *α*-enolase recognized in several pathologies and reported to date (Miles et al., 1991; Walter et al., 1995; Arza et al., 1997; Adamus et al., 1998; Simon-Nobbe et al., 2000; Pancholi 2001; Kang et al., 2008; Bruschi et al., 2014; Reed et al., 2016). Nevertheless, it is surrounded by part of the Myc binding peptide, precisely the stretches comprised by P^171^-L^191^ and T^228^-T^237^. The former also including a fragment recognized in cancer associated retinopathy and autoimmune lupus nephritis, whereas the latter is part of an epitope found in endometriosis. Therefore, K^239^ and its surrounding region seem a hot spot for adverse reactions derived from antibody recognition. Among the several enolase inhibitors available (Scatena et al., 2008; Spring and Wold 1971; Chan et al., 2016; de A S Navarro et al., 2007; Jung et al., 2013), the natural antibiotic SF2312 that mimics the carbanion intermediate of enolase reaction (Krucinska et al., 2019) and the transition state analogue phosphonoacetohydroxamic acid (PhAH) (Muller et al., 2012; Capello et al., 2016) inhibit cancer cell proliferation. However, only PhAH has been used as a therapeutic drug, although its utility to treat allergic reactions is unlikely, since the role of *α*-enolase in these outcomes seems independent of enzyme activity. Altogether, our identification of the peptide containing K^239^ as the AX-haptenation site now opens the possibility of designing new molecules to regulate its modification and/or recognition by autoantibodies with the corresponding clinical interest.

## Data Availability

Mass spectrometry data are available via ProteomeXchange with identifier PXD029712.
